# Absence of PSA Flare With Apalutamide Administered 1 Hour in Advance With GnRH Agonists: Case Report

**DOI:** 10.3389/fonc.2022.878264

**Published:** 2022-05-31

**Authors:** Zhiquan Hu, Zhenghao Liu, Zhiyuan Chen, Xing Zeng, Zhihua Wang, Chunguang Yang

**Affiliations:** ^1^ Department of Urology, Tongji Hospital Affiliated Tongji Medical College of Huazhong University of Science and Technology (HUST), Wuhan, China; ^2^ Department of Urology, Renmin Hospital of Wuhan University, Wuhan, China

**Keywords:** hormone-sensitive prostate cancer, ADT, GnRH agonist, flare, apalutamide

## Abstract

**Objective:**

To examine the effects of apalutamide on endocrine function and flare prevention in metastatic hormone-sensitive prostate cancer (mHSPC) patients administered GnRH agonists.

**Methods:**

The first newly diagnosed mHSPC patient took apalutamide for 2 weeks followed by combination with GnRH agonist, as recommended by clinical guidelines. Serum luteinizing hormone (LH), testosterone, and PSA were detected during the oral administration of apalutamide before and after ADT. Eight newly diagnosed mHSPC patients innovatively took apalutamide 1 hour before GnRH agonist administration; LH, testosterone and PSA were detected before and after ADT.

**Results:**

In the first patient, LH and testosterone levels were increased during apalutamide monotherapy, and serum PSA levels decreased rapidly, demonstrating apalutamide effectively blocked AR signaling. In patients on the 1-hour regimen, combined treatment with apalutamide and GnRH agonists led to peak level of testosterone on day 3 and castration level on day 28, while PSA decreased continuously. No one experienced dysuria or bone pain worsen after ADT.

**Conclusion:**

Taking apalutamide 1 hour in advance may effectively prevent the flare-up effect in prostate cancer patients treated with GnRH agonists. Compared with the 2-week regimen, the 1-hour regimen could simplify the treatment process and bring testosterone to castration levels in advance.

## Introduction

For metastatic hormone-sensitive prostate cancer (mHSPC), androgen deprivation therapy (ADT) is the cornerstone of systemic therapy, while GnRH agonists are the mainstream choice for ADT (1, 2). Several guidelines recommend the use of androgen receptor (AR) antagonists for 1 to 4 weeks prior to GnRH agonist injection to prevent initial flare effects (3–5). Apalutamide is a new generation of AR antagonists. Compared with first-generation AR blockers such as bicalutamide, apalutamide can block AR more efficiently and should have more advantages in preventing the ignition effect of GnRH agonists (6–8).However, the effects of apalutamide monotherapy on hormone secretion and the prevention of ignition effects have not been fully studied in clinical studies. Among the 9 newly diagnosed mHSPC patients, 1 took apalutamide for 2 weeks, then injected GnRH agonist, while 8 took apalutamide 1 hour before GnRH agonist administration. The report is as follows.

## Case Presentation

The baseline data of all patients were shown in **Table 1**. This study was approved by the Ethics Committee of Tongji Hospital, Tongji Medical College, Huazhong University of Science and Technology (TJH-IRB20211246).

**Table 1:** Basic patient information.

No.	Age	Gleason score	TNM stage	PSA (ng/ml)	LH (mIU/ml)	Testosterone (ng/ml)	Timein advance
1	55	4+3 = 7	T2cN1M1b	269.541		3.43	2 week
2	64	4+5 = 9	T4N1M1b	437.42	4.9	3.43	1h-1
3	65	4+5 = 9	T3bN1M1b	269.26	6.36	4.48	1h-2
4	65	5+4 = 9	T4N1M1b	897.51	11.45	2.45	1h-3
5	79	4+5 = 9	T3bNxM1b	77.534	3.53	3.89	1h-4
6	68	4+5 = 9	T4NxM1b	86.732	8.2	2.35	1h-5
7	75	5+4 = 9	T3bNxM1b	200.71	10.86	5.56	1h-6
8	55	4+5 = 9	T4N1M1b	98.062	2.2	2.25	1h-7
9	68	4+5 = 9	T3bN1M1b	132.22	10.85	5.1	1h-8

### 2-Week Regimen

#### Patient 1

**Figure 1:**
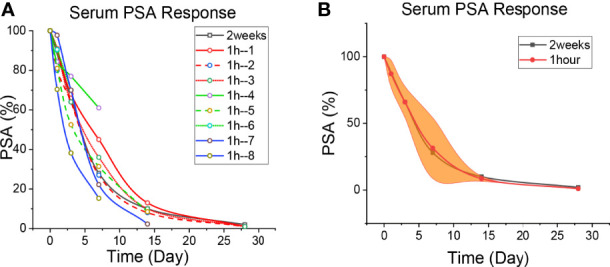
Patient’s PSA level. **(A)** The level of PSA reduction in each patient. The black line is the 2-week regimen; Red, green and blue are patients with 1H regimen. **(B)** Median reduction of PSA levels in patients with 1H regimen. The black line is the 2-week regimen; The red line is the patients with 1h regimen; The yellow areas are interquartile spacing.

**Figure 2:**
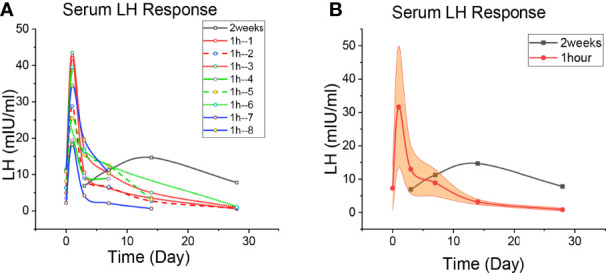
Patient’s LH level. **(A)** The level of LH change in each patient. The black line is the 2-week regimen; Red, green and blue are patients with 1H regimen. **(B)** Median changes of LH levels in patients with 1H regimen. The black line is the 2-week regimen; The red line is the patients with 1h regimen; The yellow areas are interquartile spacing.

**Figure 3:**
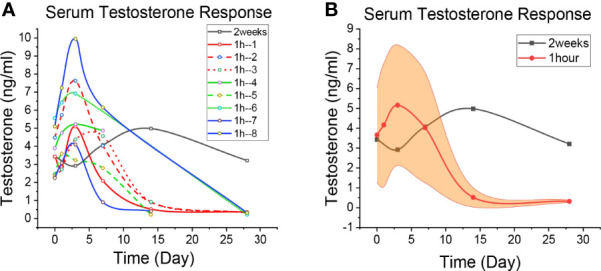
Patient’s Testosterone level. **(A)** The level of Testosterone change in each patient. The black line is the 2-week regimen; Red, green and blue are patients with 1H regimen. **(B)** Median changes of Testosterone levels in patients with 1H regimen. The black line is the 2-week regimen; The red line is the patients with 1h regimen; The yellow areas are interquartile spacing.

A 55-year-old man was hospitalized for lower extremity deep vein thrombosis and pulmonary embolism in December 2020. Screening for tumor markers found that serum PSA was 269.5ng/ml. Abdominal CT scan: Slightly enhancing low-density nodule in the left lobe of the prostate, enlarged lymph nodes adjacent to the iliac vessels on both sides (the largest one 34*30mm). Bone scan showing increased activity in right scapula, the axillary side of the right sixth rib, and the left fifth anterior rib. Prostate biopsy showed: prostate adenocarcinoma, Gleason score 4 + 3 = 7. TNM staging was considered as T2cN1M1b, stage IV. The patient had dysuria and frequent urination. Referring to the AUA guidelines and NCCN guidelines, the patient received GnRH agonist injections after 2 weeks of apalutamide treatment and continued oral apalutamide. Serum luteinizing hormone (LH) and testosterone levels at admission were 6.9mIU/ml and 3.43ng/ml, respectively. When apalutamide monotherapy for 3 days, PSA decreased by 34%. Taking into account the half-life of PSA (about 3 days), newly generated PSA in the third day is only about 16% of the original (**Supplementary Figure 1**). After 2 weeks of apalutamide treatment, LH and testosterone levels increased to 14.67 mIU/ml and 4.98 ng/ml, and PSA level decreased from 269.5 ng/mL at admission to 27.649 ng/mL (see **Figures 1**–**3** and **Supplementary Table 1**). The significant effects of apalutamide on LH, testosterone, and PSA demonstrate the high AR-binding affinity of apalutamide. After GnRH agonist (Goserelin) treatment in this patient, PSA declined steadily, while testosterone reached castration levels (41 ng/dl) after 28 days of ADT. The testosterone reached the lowest value (22 ng/dl) after 40 days of ADT. The delay in the decline of testosterone is associated with the increase in LH during apalutamide administration. Dysuria and bone pain resolved during treatment.

### 1-Hour Regimen

The baseline data of **Patient 2-9** were shown in **Table 1**. Given the higher AR-binding affinity of apalutamide, 1 hour after receiving apalutamide monotherapy, the patient was injected with GnRH agonist (Goserelin or Leuprorelin) (**Supplementary Tables 2–9**). The serum PSA, LH and testosterone levels were detected on day 0 (before treatment), 1, 3, 7, 14, and 28 (**Figures 1** **–** **3** and **Supplementary Tables** for details). PSA decreased steadily after ADT, while LH and testosterone rose to their peaks on day 1 and day 3, respectively. Testosterone reached the castration level on day 28, which was earlier than the two-week regimen. Two patients had dysuria, and four had bone pain, all of which were prostate cancer involvement. The patients had no biochemical or clinical “flare” during treatment. Symptoms such as bone pain and dysuria significantly improved in the first week of intervention.

### Methods

**2-week regimen:** Patient was treated with apalutamide 240 mg on days 0-14; combined with a GnRH agonist from day 15.

**1-hour regimen:** GnRH agonist was given combined with oral apalutamide 240 mg for 1 hour. Then, apalutamide 240mg daily and GnRH agonist once monthly as usual.

All patients were hospitalized.

## Discussion

In 2020, the number of new prostate cancer cases in the world has reached 1.4 million, ranking second among men (9). About 30% of Chinese prostate cancer patients are in a metastatic state when firstly diagnosed. ADT combined with AR antagonist therapy is one of the main treatment options (10). Apalutamide, as a synthetic biaryl thiohydantoin compound, can inhibit AR nuclear translocation, DNA binding and transcription of AR target genes (11). The SPARTAN study (12) included 1207 nmCRPC patients, and the median metastasis-free survival (MFS) after apalutamide treatment increased from 16.2 months to 40.5 months. TITAN study (6) included 1052 mHSPC patients, and showed significantly advanced in OS when taking apalutamide. Moreover, apalutamide has been approved by the FDA and CFDA for the treatment of nmCRPC and mHSPC.

During the first week after GnRH agonist injection, due to its agonistic effect on the pituitary gland, the serum LH rebounded, followed by an increase in testosterone secretion. Some patients may experience aggravation of clinical symptoms such as bone pain, spinal cord compression, and dysuria (13). Testosterone can activate AR in prostate cancer cells (14) and promote its entry into the nucleus to regulate PSA transcription, eventually causing an increase in serum PSA levels, which is called PSA flare phenomenon (15, 16). Testosterone reduces to the castration level after 4 weeks of GnRH agonist injection (3). For hormone-sensitive prostate cancer, anti-androgen drugs should be used over 1 week before the initial application of GnRH agonists to fully block AR receptors and prevent the “flare” phenomenon (4). In a number of clinical trials, different anti-androgen drugs such as nilutamide (17), estramustine phosphate (ECT) (18), chlormadinone acetate or diethylstilbestrol diphosphate (19) etc. taken 1-4 weeks in advance are effective in preventing PSA flare. This is comparable to the result of apalutamide taken 1 hour in advance in our study. In another study, patients using both Long-term ECT and goserelin acetate depot showed a slow rise in PSA levels for at least 8 weeks. Furthermore, when treated with long-term and short-term chlormadinone acetate or diethylstilbestrol diphosphate, the PSA decreased by about 70% by the end of 2 weeks, slower than 1-hour regimen.

For newly diagnosed mHSPC patients, in order to avoid PSA flare, The AUA guidelines recommend 4 weeks of antiandrogen therapy to reduce the clinical risk of “testosterone surge”; the NCCN guidelines also suggest that antiandrogen therapy should be administered prior to or concurrently with LHRH agonists and continued for at least 7 days. The effect of single use of bicalutamide on endocrine *in vivo* has been reported: LH, FSH, testosterone and dihydrotestosterone all increased to varying degrees (20). After oral administration of flutamide and bicalutamide for 4 weeks (21), the testosterone level remained high, and the PSA level decreased by about 70%, which was comparable to that of apalutamide for 1 week (**Supplementary Table 1**), suggesting that the time can be shortened when apalutamide is used to prevent the flare-up effect of GnRH agonist. Taking into account the half-life of PSA (about 3 days), newly generated PSA in the third day is only about 16% of the original (**Supplementary Figure 1**), lower than administration of flutamide and bicalutamide for 4 weeks. Moreover, after oral administration, the serum concentration of apalutamide was close to the peak concentration at 1 hour and reached the peak at 2 hours in CRPC patients (22). Similar results were seen in another study (23). This is why the GnRH agonist is applied one hour after oral administration of apalutamide in Patient 2-9.

In study LACOG 0415, the patients were divided into goserelin + abiraterone acetate + prednisone group (ADT + AAP group), apalutamide + abiraterone acetate + prednisone group (APA + AAP group) and apalutamide alone group (APA group) (24). During the 25-week follow-up period, testosterone levels in the APA group continued to rise while the other two groups remained low. This may be related to the negative feedback inhibition of testosterone on the hypothalamus and pituitary *in vivo* after the antagonist blocks AR (20). Similarly, it is shown that apalutamide can increase testosterone lastingly, but the effect on PSA and LH is still unclear, especially the changes of PSA and hormone levels within first week.

The results of a multicenter study aimed at investigating the efficacy of goserelin with or without antiandrogen drugs showed that patients on concomitant anti-androgen drugs had slower disease progression, better prognosis, and fewer PSA flares in early stages (25). Another clinical trial of leuprolide with or without nilutamide showed that patients had lower levels of prostatic acid phosphatase and lower levels of LH and testosterone elevations in combination with nilutamide (26). The above results show that GnRH agonists combined with anti-androgen drugs are more effective in controlling the levels of PSA, LH, and testosterone. As a new generation of anti-androgen drugs, apalutamide is more efficient in blocking AR receptors.

The PSA changes of the patients who underwent the 2-week regimen and the 1-h regimen are shown in **Figure 1**, and the specific values are shown in **Supplementary Tables 1–9**. It is seen that PSA decreased continuously in every patient. Taking apalutamide 1 hour in advance could efficiently prevent the PSA flare effect in prostate cancer patients treated with GnRH agonists. The declines on the third day are approximately 32% (the 2-week regimen) and 34% (the 1-h regimen), while on the 7th day, it was about 72% and 66%. The decrease in PSA on day 7 was consistent with 4 weeks of bicalutamide treatment (21), indicating a strong blocking effect of apalutamide. The half-life of PSA in human serum is approximately 3 days (27, 28). The serum PSA of the first patient on apalutamide alone for 3 days was about two-thirds of that before treatment. Given that the PSA at the beginning of treatment was reduced by half after 3 days, therefore, prostate cancer cells secrete PSA only one-sixth of the pre-treatment level after three days of oral administration of apalutamide (**Supplementary Figure 1**). This suggests that apalutamide can block AR and exert biological effects before peak serum concentration.

The LH changes of these three patients are shown in **Figure 2**, and the specific values are shown in **Supplementary Tables 1–9**. The LH of the patients in the 2-week regimen continued to rise after oral apalutamide monotherapy. One experiment showed peak LH concentrations (200% increase from baseline) on day 1 of GnRH agonist application and peak testosterone concentrations on day 3 (13). In **Figure 2**, every patient of the 1h regimen had a peak LH on day 1, which was mainly caused by the initial application of GnRH agonists; peak concentrations in 8 patients increased by approximately 408%, higher than 200%, suggesting the involvement of apalutamide. LH levels subsequently declined, and it was significantly faster for the patients of 1h regmen.

Changes in testosterone in patients who underwent the 2-week regimen and the 1-h regimen are shown in **Figure 3**, and the specific values are shown in **Supplementary Tables 1–9**. Similar to the LACOG 0415 study, patients on the 2-week regimen showed an overall upward trend within the first 14 days of apalutamide alone. But LACOG 0415 was studied on a weekly basis, ignoring data from the 3 days before the start of treatment. Patients in the 2-week regimen experienced a rapid decline in testosterone following combined GnRH agonists, reaching castration level (41 ng/dl) at day 28, then reached the lowest value (22 ng/dl) on day 40 day of ADT, showing the delay in the decline of testosterone. Meanwhile, testosterone in the 1h regimen reached castration levels on day 28 (34 ng/dl in average), indicating that the decline of testosterone was not significantly affect during ADT. Thus, the 1-hour regimen bring testosterone to castration levels in advance, compared with 2 weeks regimen.

Basic research shows that apalutamide can exert a strong AR receptor antagonistic effect in a short time, thereby reducing PSA levels. It has been reported in the literature that apalutamide can significantly inhibit the transcription level of PSA mRNA in prostate cancer cells for 16 hours *in vitro* (29). Apalutamide can effectively kill prostate tumor cells in 1 day (30). Adding excess testosterone to the 22Rv1 cell line mimics the “testosterone rebound phenomenon”, apalutamide can significantly enhance the lethality of radiotherapy and has a concentration-dependent property (31),and also significantly up-regulate the expression of AR, PSA, TMPRSS2, etc., while there is no obvious response in CRPC cell lines such as PC3 and DU145 (14). Chris Tran, the inventor of apalutamide, has already reported (11): *In vitro* cell experiments, apalutamide significantly reduced PSA mRNA levels in LNCaP/AR cells for 8 hours. In *in vitro* animal experiments, the concentration of apalutamide in the serum reached the blocking effect of AR 24 hours after oral administration of mice. The strong AR blocking ability of apalutamide was demonstrated in the 1-hour regimen cases. One hour after oral administration of apalutamide, GnRH agonist was used, and the PSA decreased steadily, indicating that the use of apalutamide for one hour can effectively block AR receptors and avoid the flare effect caused by subsequent increases in LH and testosterone.

This study revealed for the first time that apalutamide monotherapy can rapidly lower serum PSA levels, while raise LH and testosterone in mHSPC patients. Absence of PSA flare with apalutamide administered 1 hour in advance in mHSPC patients treated with GnRH agonists. Furthermore, compared with the 2-week regimen, the 1-hour regimen can bring testosterone to castration levels earlier. This may have certain reference significance for simplifying the treatment process.

## Data Availability Statement

The original contributions presented in the study are included in the article/**Supplementary Material**. Further inquiries can be directed to the corresponding author.

## Ethics Statement

The studies involving human participants were reviewed and approved by Ethics Committee of Tongji Hospital, Tongji Medical College, Huazhong University of Science and Technology. The patients/participants provided their written informed consent to participate in this study.

## Author Contributions

Conceptualization: CY. Data curation: ZH, ZW, and ZC. Formal analysis: ZH and CY. Funding acquisition: CY. Investigation: XZ and ZC. Methodology: ZH and CY. Project administration: ZH. Resources: CY. Software: XZ. Supervision: ZW. Validation: CY. Visualization: ZL. Writing – original draft: ZL and CY. Writing – review and editing: ZH. All authors contributed to the article and approved the submitted version.

## Funding

This work was supported by National Natural Science Foundation of China (grant number 81702989).

## Conflict of Interest

The authors declare that the research was conducted in the absence of any commercial or financial relationships that could be construed as a potential conflict of interest.

## Publisher’s Note

All claims expressed in this article are solely those of the authors and do not necessarily represent those of their affiliated organizations, or those of the publisher, the editors and the reviewers. Any product that may be evaluated in this article, or claim that may be made by its manufacturer, is not guaranteed or endorsed by the publisher.

## References

[1] Hussain M Fizazi K Saad F Rathenborg P Shore N Ferreira U. Enzalutamide in Men With Nonmetastatic, Castration-Resistant Prostate Cancer. N Engl J Med (2018) 378(26):2465–74. doi: 10.1056/NEJMoa1800536 PMC8288034 29949494

[2] Ryan CJ Smith MR de Bono JS Molina A Logothetis CJ de Souza P. Randomized Phase 3 Trial of Abiraterone Acetate in Men With. N Engl J Med (2013) 368(2):138–48. doi: 10.1056/NEJMoa1209096 PMC3683570 23228172

[3] Klotz L Boccon-Gibod L Shore ND Molina A Logothetis CJ Souza P. The Efficacy and Safety of Degarelix: A 12-Month, Comparative, Randomized, Open-Label, Parallel-Group Phase III Study in Patients With Prostate Cancer. BJU Int (2008) 102(11):1531–8. doi: 10.1111/j.1464-410X.2008.08183.x 19035858

[4] Cornford P van den Bergh RCN Briers E Broeck TV Cumberbatch MG Santis MD. EAU-EANM-ESTRO-ESUR-SIOG Guidelines on Prostate Cancer. Part II-2020 Update: Treatment of Relapsing and Metastatic Prostate Cancer. Eur Urol (2021) 79(2):263–82. doi: 10.1016/j.eururo.2020.09.046 33039206

[5] Dearnaley DP Syndikus IM Mossop HM Khoo V Birtle A Bloomfield D. Conventional Versus Hypofractionated High-Dose Intensity-Modulated Radiotherapy for Prostate Cancer: 5-Year Outcomes of the Randomised, non-Inferiority, Phase 3 CHHiP Trial. Lancet Oncol (2016) 17(8):1047–60. doi: 10.1016/S1470-2045(16)30102-4 PMC4961874 27339115

[6] Chi KN Agarwal N Bjartell A Chung BH Gomes AJPS Given R. Apalutamide for Metastatic, Castration-Sensitive Prostate Cancer. N Engl J Med (2019) 381(1):13–24. doi: 10.1056/NEJMoa1903307 31150574

[7] Smitha MR Antonarakisb ES Ryanc CJ Berry WR Shore ND Liu G. Phase 2 Study of the Safety and Antitumor Activity of Apalutamide(ARN-509), A Potent Androgen Receptor Antagonist, in the High-Risk Nonmetastatic Castration-Resistant Prostate Cancer Cohort. Eur Urol (2017) 70(6):963–70. doi: 10.1016/j.eururo.2016.04.023 PMC5568792 27160947

[8] Rathkopf DE Morris MJ Fox JJ Danila DC Slovin SF Hager JH. Phase I Study of ARN-509, A Novel Antiandrogen, in the Treatment of Castration-Resistant Prostate Cancer. J Clin Oncol (2013) 31:3525–30. doi: 10.1200/JCO.2013.50.1684 PMC3782148 24002508

[9] Sung H Ferlay J Siegel RL Laversanne M Soerjomataram I Jemal A Global Cancer Statistics 2020: GLOBOCAN Estimates of Incidence and Mortality Worldwide for 36 Cancers in 185 Countries. CA: Cancer J Clin (2021) 71(3):209–49. doi: 10.3322/caac.21660 33538338

[10] Kyriakopoulos CE Chen YH Carducci MA Liu G Jarrard DF Hahn NM. Chemohormonal Therapy in Metastatic Hormone-Sensitive Prostate Cancer: Long-Term Survival Analysis of the Randomized Phase III E3805 CHAARTED Trial. J Clin Oncol (2018). doi: 10.1200/JCO.2017.75.3657 PMC5891129 29384722

[11] Clegg NJ Wongvipat J Joseph JD Tran C Ouk S Dilhas A. ARN-509a Novel Antiandrogen for Prostate Cancer Treatment. Cancer Res (2012) 72(6):1494–503. doi: 10.1158/0008-5472.CAN-11-3948 PMC3306502 22266222

[12] Smith MR Saad F Chowdhury S Oudard S Hadaschik BA Graff JN. Apalutamide Treatment and Metastasis-Free Survival in Prostate Cancer. N Engl J Med (2018) 378(15):1408–18. doi: 10.1056/NEJMoa1715546 29420164

[13] Thompson IM. Flare Associated With LHRH-Agonist Therapy. Rev Urol (2001) 3(Suppl 3):S10–4. PMC1476081 16986003

[14] Koukourakis MI Kakouratos C Kalamida D Mitrakas A Pouliliou S Xanthopoulou E. Comparison of the Effect of the Antiandrogen Apalutamide (ARN-509) Versus Bicalutamide on the Androgen Receptor Pathway in Prostate Cancer Cell Lines. Anticancer Drugs (2018) 29(4):323–33. doi: 10.1097/CAD.0000000000000592 29381490

[15] Ueda T Shiraishi T Ito S Ohashi M Matsugasumi T Yamada Y. Abiraterone Acetate Versus Bicalutamide in Combination With Gonadotropin Releasing Hormone Antagonist Therapy for High Risk Metastatic Hormone Sensitive Prostate Cancer. Sci Rep (2021) 11(1):10094. doi: 10.1038/s41598-021-89609-2 33980956 PMC8115638

[16] Miller K Simson G Goble S Persson B. Efficacy of Degarelix in Prostate Cancer Patients Following Failure on Luteinizing Hormone-Releasing Hormone Agonist Treatment: Results From an Open-Label, Multicentre, Uncontrolled, Phase II Trial (CS27). Ther Adv Urol (2015) 7(3):105–15. doi: 10.1177/1756287215574479 PMC4485413 26161141

[17] Kuhn JM T Billebaud HN Moulonguet A Louis JF Costa P Husson JM. Prevention of the Transient Adverse Effects of a Gonadotropin-Releasing Hormone Analogue (Buserelin) in Metastatic Prostatic Carcinoma by Administration of an Antiandrogen (Nilutamide). N Engl J Med (1989) 321(7):413–8. doi: 10.1056/NEJM198908173210701 2503723

[18] Shimizu TS Shibata Y Jinbo H Satoh J Yamanaka H. Estramustine Phosphate for Preventing Flare-Up in Luteinizing Hormone-Releasing Hormone Analogue Depot Therapy. Eur Urol (1995) 27(3):192–5. doi: 10.1159/000475159 7541359

[19] Kotake T Usaml M Akaza H Koiso K Homma Y Kawabe K. Goserelin Acetate With or Without Antiandrogen or Estrogen in the Treatment of Patients With Advanced Prostate Cancer: A Multicenter, Randomized, Controlled Trial in Japan. Jpn J Clin OncoI (1999) 29(11):562–70. doi: 10.1093/jjco/29.11.562 10678560

[20] Verhelst J Denis L Van Vliet P Van Poppel H Braeckman J Van Cangh P. Endocrine Profiles During Administration of the New non-Steroidal Anti-Androgen Casodex in Prostate Cancer. Clin Endocrinol (Oxford) (1994) 41(4):525–30. doi: 10.1111/j.1365-2265.1994.tb02585.x 7525125

[21] Nakai Y Tanaka N Anai S Miyake M Tatsumi Y Fujimoto KA. A Randomized Control Trial Comparing the Efficacy of Antiandrogen Monotherapy: Flutamide vs. Bicalutamide. Hormones Cancer (2015) 6(4):161–7. doi: 10.1007/s12672-015-0226-1 PMC10355925 26024831

[22] Belderbos B Wit R Chien C Mitselos A Hellemans P Jiao J. An Open-Label, Multicenter, Phase Ib Study Investigating the Effect of Apalutamide on Ventricular Repolarization in Men With Castration-Resistant Prostate Cancer. Cancer Chemother Pharmacol (2018) 82(3):457–68. doi: 10.1007/s00280-018-3632-6 PMC6105166 29974203

[23] Pang X Wang Y Chen Y. Design, Synthesis, and Biological Evaluation of Deuterated Apalutamide With Improved Pharmacokinetic Profiles. Bioorganic Medicinal Chem Lett (2017) 27(12):2803–6. doi: 10.1016/j.bmcl.2017.04.071 28478926

[24] Maluf FC Schutz FA Cronemberger EH Luz MA Martins SPS Muniz DQB. A Phase 2 Randomized Clinical Trial of Abiraterone Plus ADT, Apalutamide, or Abiraterone and Apalutamide in Patients With Advanced Prostate Cancer With non-Castrate Testosterone Levels (LACOG 0415). Eur J Cancer (2021) 158:63–71. doi: 10.1016/j.ejca.2021.08.032 34655838

[25] Kotake T Usam M Akaza H Koiso K Homma Y Kawabe K. Goserelin Acetate With or Without Antiandrogen or Estrogen in the Treatment of Patients With Advanced Prostate Cancer: A Multicenter, Randomized, Controlled Trial in Japan. Jpn J Clin OncoI (1999) 29(11):562–70. doi: 10.1093/jjco/29.11.562 10678560

[26] Kuhn JM Billebaud T Navratil H Moulonguet a Fiet J Grise P. Prevention of the Transient Adverse Effects of A Gonadotropin-Releasing Hormone Analogue (Buserelin) in Metastatic Prostatic Carcinoma by Administration of an Antiandrogen (Nilutamide). N Engl J Med (1989) 321(7):413–8. doi: 10.1056/NEJM198908173210701 2503723

[27] Carobene A Guerra E Locatelli M Cucchiara V Briganti A Aarsand AK. Biological Variation Estimates for Prostate Specific Antigen From the European Biological Variation Study; Consequences for Diagnosis and Monitoring of Prostate Cancer. Clin Chim Acta (2018) 486):185–91. doi: 10.1016/j.cca.2018.07.043 30063887

[28] Martin B Cheli C Davis R Ward M Kokatnur M Mercante D. cPSA and fPSA Elimination in African-American Men. Prostate Cancer Prostatic Dis (2003) 6:163–8. doi: 10.1038/sj.pcan.4500649 12806377

[29] Liu C Armstrong CM Ning S Yang JC Lou W Lombard AP. ARVib Suppresses Growth of Advanced Prostate Cancer via Inhibition of Androgen Receptor Signaling. Oncogene (2021) 40(35):5379–92. doi: 10.1038/s41388-021-01914-2 PMC8413131 34272475

[30] Eberli D Kranzbuhler B Mortezavi A Sulser T Salemi S. Apalutamide in Combination With Autophagy Inhibitors Improves Treatment Effects in Prostate Cancer Cells. Urol Oncol (2020) 38(8):619–83. doi: 10.1016/j.urolonc.2020.04.030 32466878

[31] Kakouratos C Kalamida D Lamprou I Xanthopoulou E Nanos C Giatromanolaki A . Apalutamide Radio-Sensitisation of Prostate Cancer. Br J Cancer (2021) 125(10):1377–87. doi: 10.1038/s41416-021-01528-1 PMC8575888 34471256

