# Supplementary material for: Absence of PSA Flare With Apalutamide Administered 1 Hour in Advance With GnRH Agonists: Case Report
Source: Front Oncol. 2022 May 31;12:878264. doi: 10.3389/fonc.2022.878264 (PMC9193224; doi:10.3389/fonc.2022.878264)
Supplement: Supplementary file 1 [file DataSheet_1.docx]

**Supplement Figure 1**. Newly generated PSA within 3 days of apalutamide monotherapy.

The blue area represents the total amount of PSA present on day 0, and the green area represents the newly generated amount within 3 days. PSA degrades 50% in 3 days.

**Supplement Table 1**. 2-week regimen patient follow-up data

|  | PSA（ng/ml） |  | LH（mIU/ml) |  | testosterone（ng/ml) |  |
| --- | --- | --- | --- | --- | --- | --- |
| Day 0： | 269.541 |  | # |  | 3.43 |  |
| Day 3： | 179.001 | -34% | 6.9 |  | 2.92 | -15% |
| Day 7： | 76.07 | -72% | 11.23 |  | 4.07 | +19% |
| Day 14： | 27.649 | -90% | 14.67 |  | 4.98 | +45% |
| + ADT |  |  |  |  |  |  |
| Day 14： | 5.32 |  | 7.8 |  | 3.21 |  |
| Day 28: | 1.034 | -99.6% | 1.01 |  | 0.41 | -88% |
| Day 40： | 0.198 | -99.9% | 0.26 |  | 0.22 | -94% |

Day 0 = before treatment; +ADT = combine with ADT treatments; PSA = prostate specific androgen; LH = Luteinizing hormone.

**Supplement Table 2**. Follow-up data of the first 1-h regimen patient

(1h-1)

|  | PSA（ng/ml) |  | LH(mIU/ml) |  | testosterone（ng/ml) |  |
| --- | --- | --- | --- | --- | --- | --- |
| Day 0 | 437.42 |  | 4.9 |  | 3.43 |  |
| +ADT |  |  |  |  |  |  |
| Day 1 | 399.1 | -9% | 42 | 757% | 2.85 | -17% |
| Day 3 | 296.28 | -32% | 10.49 | 114% | 5.1 | 49% |
| Day 7 | 198.07 | -55% | 6.32 | 29% | 2.09 | -39% |
| Day 14 | 52.18 | -87% | 3.53 | -28% | 0.52 | -85% |
| Day 28 | 3.68 | -99% | 0.53 | -89% | 0.36 | -90% |

Day 0 = before treatment; +ADT = combine with ADT treatments; PSA = prostate specific androgen; LH = Luteinizing hormone.

A 64-year-old man developed pelvic pain without obvious incentives, without hematuria. He was admitted to the hospital for examination on November 3, 2021. Screening for tumor markers revealed a serum PSA of 437.42ng/ml. mpMRI scan of the prostate showed that the prostate mass broke through the capsule, with the left seminal vesicle involved. PI-RADS score: 5; bilateral acetabulum, pubis, right ischium, slightly longer T2 signal foci on the left side of the sacrum metastasized; local thickening of the rectal wall. Bone scan results showed that the occipital bone, right scapula, thoracic vertebra T6, sacral vertebra, right ilium-right acetabulum-right ischium and right upper femur saw radioactive shadows，mild radioactive shadows were seen between the left femoral trochanter, and bone metastasis was considered. Prostate biopsy: prostate adenocarcinoma, Gleason score: 4+5=9, WHO/ISUP grading grouping: 5. TNM stage T4N1M1b (stage IV, high tumor volume). On admission, the endocrine indexes LH and testosterone were 4.9mIU/ml and 3.43ng/ml, respectively. Given the stronger AR blocking ability of apalutamide, 1 hour after receiving apalutamide monotherapy, the patient was injected with a Goserelin and continued with oral apalutamide, the serum PSA, LH and testosterone levels were detected on the 0th (before treatment), 1, 3, 7, 14, and 28 days (see Figure 1，2，3 and Supplement Table 2 for details). PSA decreased steadily from day one, LH and testosterone rose to their peaks on the first and third days, respectively. Among them, testosterone reached the castration level at 28 days, which was about 12 days earlier than the two-week regimen. The patient had no biochemical or clinical "flare" during treatment, and symptoms of bone pain and dysuria improved significantly on day 3 of drug treatment.

**Supplement Table 3**. Follow-up data of the second 1-h regimen patient

(1h-2)

|  | PSA（ng/ml) |  | LH(mIU/ml) |  | testosterone（ng/ml) |  |
| --- | --- | --- | --- | --- | --- | --- |
| Day 0 | 269.26 |  | 6.36 |  | 4.48 |  |
| +ADT |  |  |  |  |  |  |
| Day 1 | 215.71 | -20% | 28.77 | 352% | 5.73 | 28% |
| Day 3 | 171.83 | -36% | 9.12 | 43% | 7.63 | 70% |
| Day 7 | 71.693 | -73% | 6.62 | 4% | 4.03 | -10% |
| Day 14 | 20.59 | -92% | 2.71 | -57% | 0.9 | -80% |
| Day 28 | 2.03 | -99% | 0.54 | -89% | 0.36 | -92% |

Day 0 = before treatment; +ADT = combine with ADT treatments; PSA = prostate specific androgen; LH = Luteinizing hormone.

A 65-year-old man was admitted in hospital for dysuria for one week. Total PSA = 269.26ng/ml. mpMRI scan of the prostate: neoplastic lesions of prostate with invasion of bilateral seminal vesicles, and the possibility of bilateral pelvic and pararectal lymph node metastasis; abnormal signal nodule on the left iliac bone with limited diffusion and possible metastasis. Bone scan showed that the radioactive shadow was seen in the axillary segment of the sixth rib on the left side, and the possibility of tumor metastasis was considered. Prostate biopsy showed prostate adenocarcinoma, Gleason score 4+5=9, WHO/ISUP grading grouping: 5. TNM stage T3bN1M1b (stage IV, low tumor volume). Similar to the second patient, 1 hour after receiving apalutamide monotherapy, the patient was injected with a Goserelin and continued with oral apalutamide, serum PSA, LH and testosterone levels were collected on days 0 (before treatment), 1, 3, 7, 14 and 28 (see Figure 1，2，3 and Supplement Table 3 for details). PSA dropped by 36% on the third day, and LH and testosterone rose to a peak on the first and third days, respectively. And the patient's symptoms of dysuria improved, with no biochemical or clinical "flare" during treatment.

**Supplement Table 4**. Follow-up data of the third 1-h regimen patient

(1h-3)

|  | PSA（ng/ml) |  | LH(mIU/ml) |  | testosterone（ng/ml) |  |
| --- | --- | --- | --- | --- | --- | --- |
| Day 0 | 897.51 |  | 11.45 |  | 2.45 |  |
| +ADT |  |  |  |  |  |  |
| Day 1 | 810.47 | -10% | 43.49 | 380% | 2.72 | 111% |
| Day 3 | 572.27 | -36% | 16.97 | 148% | 4.38 | 179% |
| Day 7 | 324.55 | -64% | 10.33 | 90% | 4.57 | 187% |
| Day 14 | 91.604 | -90% | 5.02 | 44% | 0.92 | 38% |
| Day 28 | 6.392 | -99% | 0.95 | 8% | 0.32 | 13% |

Day 0 = before treatment; +ADT = combine with ADT treatments; PSA = prostate specific androgen; LH = Luteinizing hormone.

A 65-year-old man complained of dysuria and bone pain for more than 1 month, Total PSA > 100ng/ml, CT showed enlargement of prostate accompanied by calcification, and prostate biopsy showed adenocarcinoma of prostate, Gleason score 5+4=9. After admission, relevant examination was completed: Luteinizing hormone (LH) 11.45 mIU/mL. Total prostate antigen (TPSA) >100.000 ng/mL, total prostate antigen (after dilution) 897.51 ng/mL. PET/CT examination results: 1. Prostate cancer involving bladder and bilateral seminal vesicles, retroperitoneum and multiple pelvic lymph node metastases; 2. Uneven bone density of bilateral humerus, sternum, multiple ribs, some thoracolumbar vertebrae, pelvic bones and bilateral femurs. Patients were given apatamine and Goserelin. The trend of serum PSA, LH and testosterone levels in the follow-up was consistent with that in the previous patients (see Figure 1，2，3 and Supplement Table 4 for details).

**Supplement Table 5**. Follow-up data of the fourth 1-h regimen patient

(1h-4)

|  | PSA（ng/ml) |  | LH(mIU/ml) |  | testosterone（ng/ml) |  |
| --- | --- | --- | --- | --- | --- | --- |
| Day 0 | 77.534 |  | 3.53 |  | 3.89 |  |
| +ADT |  |  |  |  |  |  |
| Day 1 | 65.318 | -16% | 19.43 | 550% | 4.74 | 122% |
| Day 3 | 59.663 | -23% | 9.62 | 273% | 5.22 | 134% |
| Day 7 | 47.155 | -39% | 8.87 | 251% | 4.87 | 125% |

Day 0 = before treatment; +ADT = combine with ADT treatments; PSA = prostate specific androgen; LH = Luteinizing hormone.

A 65-year-old man admitted to hospital due to "prostate tumor found for 1 month". Total PSA 77.534 ng/mL; Bone scan: the radioactive shadow of thoracic vertebra. mpMRI scan of the prostate: prostate hyperplasia; Abnormal patchy signals with limited diffusion in bilateral transitional and peripheral prostate zones (34mm*23mm) were considered as neoplastic lesions. PI-RADS score: 4, and bilateral seminal vesicle glands may be involved. Prostate biopsy: prostate adenocarcinoma, Gleason score: 4+5=9 points; WHO/ISUP Classification: 5. The trend of serum PSA, LH and testosterone levels in the follow-up patients was consistent with that in the previous patients (see Figure 1，2，3 and Supplement Table 5 for details).

**Supplement Table 6**. Follow-up data of the fifth 1-h regimen patient

(1h-5)

|  | PSA（ng/ml) |  | LH(mIU/ml) |  | testosterone（ng/ml) |  |
| --- | --- | --- | --- | --- | --- | --- |
| Day 0 | 86.732 |  | 8.2 |  | 2.35 |  |
| +ADT |  |  |  |  |  |  |
| Day 1 | 70.315 | -19% | 25.5 | 311% | 3.59 | 153% |
| Day 3 | 45.483 | -48% | 15.44 | 188% | 3.23 | 137% |
| Day 7 | 27.2 | -69% | 12.44 | 152% | 2.79 | 119% |
| Day 14 | 7.34 | -92% | 3.16 | -61% | 0.22 | 9% |

Day 0 = before treatment; +ADT = combine with ADT treatments; PSA = prostate specific androgen; LH = Luteinizing hormone.

A 68-year-old man with pain in the right hip area and difficulty in walking. mpMRI scan of the prostate showed occupying lesions in prostate. PSA: 86.732ng/ml. Bone scan showed multiple bone metastases throughout the body. Prostate biopsy: 5+4=9; WHO/ISUP Classification: 5. Treated with apalutamide and Goserelin. The trend of serum PSA, LH and testosterone levels in the follow-up patients was consistent with that in the previous patients (see Figure 1，2，3 and Supplement Table 6 for details).

**Supplement Table 7**. Follow-up data of the sixth 1-h regimen patient

(1h-6)

|  | PSA（ng/ml) |  | LH(mIU/ml) |  | testosterone（ng/ml) |  |
| --- | --- | --- | --- | --- | --- | --- |
| Day 0 | 200.71 |  | 10.86 |  | 5.56 |  |
| +ADT |  |  |  |  |  |  |
| Day 1 | 181.52 | -10% | 39.5 | 364% | 6.42 | 115% |
| Day 3 | 140.24 | -30% | 16.45 | 151% | 6.92 | 124% |
| Day 28 | 3.17 | -98% | 1.12 | 10% | 0.23 | 4% |

Day 0 = before treatment; +ADT = combine with ADT treatments; PSA = prostate specific androgen; LH = Luteinizing hormone.

A 75-year-old man hospitalized due to "1-week elevated PSA". mpMRI scan of the prostate: 1. Prostate tumor tendency, invasion of bladder and seminal vesicle; 2. Pelvis and sacral caudvertebra, multiple bone destruction of femur on both sides; 3. Pelvic and bilateral lymph node enlargement. Related examination: PSA: 200.71ng/ml. Prostate biopsy: prostate adenocarcinoma, Gleason score: 5+4=9; WHO/ISUP Classification: 5. Whole body bone imaging: extensive bone metastases. Treated with apalutamide and Leuprorelin. The trend of serum PSA, LH and testosterone levels in the follow-up patients was consistent with that in the previous patients (see Figure 1，2，3 and Supplement Table 7 for details).

**Supplement Table 8**. Follow-up data of the seventh 1-h regimen patient

(1h-7)

|  | PSA（ng/ml) |  | LH(mIU/ml) |  | testosterone（ng/ml) |  |
| --- | --- | --- | --- | --- | --- | --- |
| Day 0 | 98.062 |  | 2.2 |  | 2.25 |  |
| +ADT |  |  |  |  |  |  |
| Day 1 | 95.767 | -2% | 18.31 | 832% | 3.08 | 137% |
| Day 3 | 68.054 | -31% | 4.2 | 191% | 4.1 | 182% |
| Day 7 | 21.8 | -78% | 2.13 | 97% | 0.9 | 40% |
| Day 14 | 2.248 | -98% | 0.63 | 29% | 0.41 | 18% |

Day 0 = before treatment; +ADT = combine with ADT treatments; PSA = prostate specific androgen; LH = Luteinizing hormone.

A 55-year-old man was admitted to hospital due to "1 week elevated PSA ". PSA: 98.062 ng/ml. Whole-body PSMA PET/CT imaging showed malignant neoplastic lesions of the prostate, involving the bladder and seminal vesicles, and bilateral pelvic lymph node metastasis. mpMRI scan of the prostate: irregular soft tissue mass, protruded into the bladder, and the boundary between it and the bladder wall was not clear; bilateral pelvic wall lymph nodes slightly enlarged, high signal shadow seen on DWI. Prostatic biopsy: prostate adenocarcinoma,Gleason score: 4+5=9; WHO/ISUP Classification group: 5. The patient was given apalutamide and Goserelin. The trend of serum PSA, LH and testosterone levels in the follow-up patients was consistent with that in the previous patients (see Figure 1，2，3 and Supplement Table 8 for details).

**Supplement Table 9**. Follow-up data of the eighth 1-h regimen patient

(1h-8)

|  | PSA（ng/ml) |  | LH(mIU/ml) |  | testosterone（ng/ml) |  |
| --- | --- | --- | --- | --- | --- | --- |
| Day 0 | 132.22 |  | 10.85 |  | 5.1 |  |
| +ADT |  |  |  |  |  |  |
| Day 1 | 92.97 | -30% | 34.51 | 318% | 7.24 | 142% |
| Day 3 | 50.43 | -62% | 19.56 | 180% | 9.95 | 195% |
| Day 7 | 20.13 | -85% | 10.36 | 95% | 6.15 | 121% |
| Day 28 | 2.05 | -98% | 0.43 | 4% | 0.36 | 7% |

Day 0 = before treatment; +ADT = combine with ADT treatments; PSA = prostate specific androgen; LH = Luteinizing hormone.

A 68-year-old man, admitted to hospital for "dysuria for 1 week", and with symptoms of pelvic pain. Total PSA: 132.22 ng/ml. mpMRI scan of the prostate showed: soft tissue mass with seminal vesicle involvement, bilateral iliac paravascular lymph node enlargement; Bone scan: three bone foci in the pelvis; TNM stage T3bN1M1b. Prostate biopsy results showed: prostate adenocarcinoma, Gleason score 4+5=9.Patients were given apalutamide and Leuprorelin. The trend of serum PSA, LH and testosterone levels in the follow-up patients was consistent with that in the previous patients (see Figure 1，2，3 and Supplement Table 9 for details).
